# The pre- and post-COVID-19 pandemic dengue fever patterns in southeastern coastal China in 2019 and 2024: molecular evolution and strain replacement

**DOI:** 10.3389/fmicb.2025.1607085

**Published:** 2025-08-08

**Authors:** Tianran Zhang, Yaqi Shi, Leyi Zhang, Jian Wang, Chengchao Yu, Binbin Lv, Sisi Wu, Yiru Huang, Xuewei Liu, Hupiao Dai, Mingshi Zhou, Yanjuan Liao, Wei Wang, Guankai Lin

**Affiliations:** ^1^Wenzhou Municipal Center for Disease Control and Prevention, Wenzhou Municipal Institute of Health Supervision, Wenzhou, China; ^2^Yunnan Animal Health Supervision Institute, Kunming, China; ^3^Guangxi Key Laboratory of Polysaccharide Materials and Modification, School of Marine Sciences and Biotechnology, Guangxi Minzu University, Nanning, China; ^4^The Humanities Laboratory for Ecological Civilization and Environmental Governance of Zhejiang Province, Institute of Virology, Wenzhou University, Wenzhou, China

**Keywords:** dengue virus type 1, amino acid substitutions, vaccine compatibility, molecular evolution, COVID-19

## Abstract

**Background:**

The evolutionary dynamics of dengue virus type 1 (DENV-1) in non-endemic settings such as southeastern coastal China where outbreaks predominantly stem from imported cases, remains insufficiently defined, particularly in relation to lineage displacement dynamics.

**Methods:**

Ninety-three DENV-1 isolates (56 from 2019, 37 from 2024) collected in southeastern coastal China underwent whole-genome sequencing. Phylogenetic relationships were inferred using maximum likelihood methods under the GTR + G + I model. Selection pressures were assessed through FEL, MEME, SLAC, and FUBAR. Recombination was analyzed using seven detection algorithms implemented in RDP4. Hamming distances were used to profile amino acid substitutions. Epitope mapping was performed by comparative alignment against CYD-TDV and TAK-003 vaccine reference strains.

**Results:**

Phylogenetic inference placed all isolates within genotype I, yet 2019 and 2024 sequences segregated into distinct clades: 1I_E.1 and 1I_K.2, respectively. Codon-level analyses consistently indicated purifying selection. Eighteen recombination events were identified, predominantly involving strains of Vietnamese and Cambodian origin. A total of 40 non-synonymous substitutions were conserved across both periods, while 24 mutations were exclusive to 2024 isolates, with marked enrichment in NS3 and NS5 proteins. Epitope analyses revealed 9 and 17 antigenic variants within neutralizing domains of CYD-TDV and TAK-003, respectively.

**Conclusion:**

Complete genotype turnover of DENV-1 occurred in southeastern coastal China between 2019 and 2024, driven by displacement of lineage 1I_E.1 by 1I_K.2, with implications for local transmission patterns. Observed antigenic divergence between temporal isolates emphasizes the importance of sustained genomic monitoring and targeted intervention strategies tailored to circulating strains in this region.

## 1 Introduction

Dengue fever currently places approximately 40% of the global population in endemic regions at risk, with an estimated 390 million infections annually (Wilder-Smith et al., 2019). Over recent decades, its global burden has intensified, expanding from historically endemic zones into previously unaffected areas, driven by urbanization, globalization, inadequate vector control, and climate change (Wilder-Smith et al., 2019; Messina et al., 2019). Epidemiological monitoring reveals cyclical outbreak trends modulated by herd immunity, vector ecology, and climate variability (Lourenço and Recker, 2013). While southeastern coastal China previously classified dengue as an imported condition, recent surveillance indicates a growing propensity for sustained autochthonous transmission (Wu et al., 2010), with large-scale outbreaks documented in 2019 and 2024. Transmission and viral evolution are shaped by seasonal fluctuations, intrinsic viral dynamics, and human mobility networks. Dengue virus undergoes periodic lineage replacements approximately every 4–6 years (Chen and Vasilakis, 2011). These evolutionary cycles, in conjunction with increased international travel and shifting local transmission patterns, continuously redefine the region’s dengue epidemiology. Belonging to the Flaviviridae family, dengue virus includes four serotypes (DENV-1 to DENV-4), with DENV-1 exhibiting widespread distribution across tropical and subtropical zones. The envelope (E) protein, as the principal antigenic determinant for neutralizing antibodies, constitutes a key target for vaccine strategies (Roehrig, 2003). Structurally, the E protein comprises three domains EDI, EDII, and EDIII with EDIII being integral to receptor recognition and containing epitopes conferring serotype specificity (Rey et al., 1995).

Currently available dengue vaccines include Sanofi Pasteur’s CYD-TDV (Dengvaxia^®^) and Takeda’s TAK-003 (Qdenga^®^), which adopt distinct technological platforms and strain compositions (Capeding et al., 2014; Biswal et al., 2019). CYD-TDV utilizes a live attenuated chimeric design incorporating a yellow fever virus backbone combined with dengue structural proteins, whereas TAK-003 employs an attenuated DENV-2 backbone (Guy et al., 2011). Their protective efficacy varies by serotype, highlighting concerns regarding antigenic mismatch between vaccine strains and locally circulating viruses (Wilder-Smith, 2020).

Although dengue poses an escalating public health threat in southeastern coastal China, molecular insights into DENV-1 evolution in this region remain limited. While isolated outbreaks have been examined, longitudinal genomic surveillance including interannual viral population shifts is lacking. In non-endemic regions such as southeastern coastal China, where viral transmission predominantly arises through case importation rather than sustained local circulation, the evolutionary patterns of DENV-1 remain unclear. This gap constrains understanding of region-specific arboviral adaptation trajectories. Moreover, although recent regulatory approval of dengue vaccines has marked a critical milestone, antigenic congruence between vaccine strains and locally circulating DENV-1 lineages has yet to be assessed, thereby impeding accurate projections of vaccine performance within this population.

This study aims to investigate the molecular features and evolutionary trajectories of DENV-1 strains circulating in southeastern coastal China from 2019 to 2024, utilizing whole-genome sequencing and bioinformatic analyses. Temporal dynamics of DENV-1 transmission and genetic variation are examined, and epitope-based comparisons between regional and vaccine strains are conducted to inform data-driven strategies for optimizing immunization efforts.

## 2 Materials and methods

### 2.1 RNA extraction and sequencing library construction

A total of 152 serum samples were collected from patients in southeastern coastal China in 2019, along with 60 additional samples obtained in 2024. Post-screening, only DENV-1 serotype samples with cycle threshold (CT) values below 28 were retained, while samples associated with other dengue serotypes or CT values exceeding the threshold were excluded due to inadequate quality. Based on these criteria, 132 samples from 2019 and 49 from 2024 were deemed suitable for downstream analysis. Total RNA was extracted using the RNeasy Mini Kit (Qiagen, Hilden, Germany) in accordance with the manufacturer’s protocol. Complementary DNA was synthesized via reverse transcription, followed by sequencing library construction using the Illumina DNA Prep kit (Illumina, San Diego, CA, United States). Sequencing was subsequently conducted on the Illumina MiSeq platform employing the V2 300 cycle chemistry kit.

### 2.2 Sequence assembly and genome analysis

Raw sequencing data were processed using a standardized bioinformatics pipeline. Reads with Phred scores <Q30 were excluded to maintain high assembly quality. Sequencing depth ranged from 4,410 × to 6,324 × (mean: 5,415×). Minimap2 facilitated alignment against reference genomes, followed by sequence processing and sorting via Samtools. Variant calling employed both GATK and FreeBayes, while SPAdes was applied for virus-specific *de novo* assembly. Genome coverage surpassed 99% across all samples. To limit analytical bias, sequences sharing >99.5% identity from the same residential areas were excluded, reducing potential epidemiological linkage and sampling redundancy in phylogenetic reconstruction. Complete genome sequences were obtained for all 93 samples (56 from 2019, 37 from 2024), forming the core dataset for downstream analyses.

### 2.3 Genotyping and phylogenetic analysis

To generate a robust reference dataset, custom Python scripts were employed to extract 1,600 DENV-1 envelope gene sequences from the NCBI GenBank database. Following stringent quality control, 1,381 high-confidence sequences were retained. Redundancies were eliminated using HYPHY (Pond et al., 2005), yielding a curated set of 882 non-duplicated reference sequences based on the E gene. These were subsequently aligned with sequences obtained from regional samples. Phylogenetic reconstruction was conducted using RAxML (Stamatakis, 2014) under the GTR + G + I substitution model, with 1,000 bootstrap replicates to evaluate the robustness of the tree topology. The DENV-1 prototype strain Hawaii-1944, isolated in 1944, was designated as the outgroup to define the root of the phylogenetic tree. To enhance visualization, a pruning algorithm was implemented via the ape package in R (v4.0.5), filtering nodes based on Most Recent Common Ancestor (MRCA) criteria, retaining only reference sequences sharing MRCA nodes with fewer than 50 terminal branches associated with local isolates. Genotypic and lineage assignments were conducted using the Dengue Virus Typing Tool (v4.2), which applies standardized nomenclature and genetic distance thresholds in conjunction with phylogenetic structure.

### 2.4 Selection pressure and recombination analysis

Selection pressure was assessed using HYPHY (Pond et al., 2005) via four analytical frameworks: Fixed Effects Likelihood (FEL), Mixed Effects Model of Evolution (MEME), Single Likelihood Ancestor Counting (SLAC), and Fast Unconstrained Bayesian Approximation (FUBAR). Codon sites were classified as undergoing selection if they demonstrated posterior probability >0.9 in FUBAR and *p*-values <0.05 in FEL, MEME, and SLAC outputs.

For recombination assessment, 2,000 temporally stratified DENV-1 full-genome sequences (2014–2024) were retrieved from NCBI to capture global genetic variability. These sequences were merged with locally sequenced samples and processed using RDP4 (Martin et al., 2015), incorporating seven algorithms: RDP, GENCONV, BootScan, MaxChi, Chimaera, SiScan, and 3Seq. Recombination events were deemed robust when identified by at least four methods, each yielding *p*-values <0.05.

### 2.5 Amino acid substitution analysis

Viral sequences were categorized into 2019 and 2024 cohorts and aligned against the DENV-1 reference strain (KM204119, Hawaii-1944) to identify amino acid substitutions across the polyprotein. Variant sites exhibiting sequence divergence exceeding 50% and resulting in non-synonymous mutations were prioritized for downstream analysis based on calculated Hamming distances.

### 2.6 Epitope analysis and vaccine strain comparison

Dengue virus epitopes experimentally validated and archived in the Immune Epitope Database (IEDB) were screened using Response Frequency (RF) scores, selecting immunodominant epitopes with RF >0.25 according to criteria from established evolutionary studies (Jagtap et al., 2023). Regional viral strains were compared to CYD-TDV and TAK-003 vaccine strains by computing amino acid-level differences through Hamming distance analysis. The envelope protein dimer was modeled via homology modeling on the SWISS-MODEL server (Waterhouse et al., 2018), utilizing templates optimized by global model quality and QMEAN metrics. Residues with >50% sequence divergence were classified as key variation points and subsequently visualized on the envelope protein structure using PyMOL (v2.4.1), referencing the DENV-1 E protein crystal structure (PDB ID: 8y3j).

### 2.7 Interrupted time series analysis

Interrupted time series analysis was applied to evaluate the influence of COVID-19-related interventions on dengue incidence using monthly case data spanning January 2018 to December 2024. The onset of COVID-19 control measures (January 2020) and their subsequent relaxation (December 2022) were designated as intervention points. Analysis employed the “itsa” command in Stata version 17.0 [itsa cases, single treat (1) trperiod (2020-01; 2022-12) lag (1) posttrend figure] following the approach outlined by Bernal et al. (2017). The Cumby-Huizinga test was used to assess residual autocorrelation. The segmented regression model was specified as:


$$\begin{array}{l} y\_t=\beta_{0}+\beta ₁T\_t+\beta ₂X\_t+\beta ₃X\_\mathrm{tT}\_t+\\ \beta ₄Z\_t+\beta 5Z\_\mathrm{tT}\_t+\varepsilon \_t \end{array}$$

where *y*_*t* denotes log-transformed dengue case counts at time *t*; *T*_*t* captures the time elapsed since the beginning of the study period; *X*_*t* is a binary indicator for the implementation phase of COVID-19 control measures; *X*_*tT*_*t* denotes the post-implementation time trend; *Z*_*t* indicates the period following policy relaxation; and *Z*_*tT*_*t* represents the post-relaxation trend. Corresponding coefficients quantify: *β*_0_ (initial level), *β*₁ (pre-intervention trajectory), *β*₂ (level change post-implementation), *β*₃ (slope change during intervention), *β*₄ (level change post-relaxation), and *β*₅ (slope change following relaxation).

## 3 Results

### 3.1 Phylogenetic analysis

**Figure 1:**
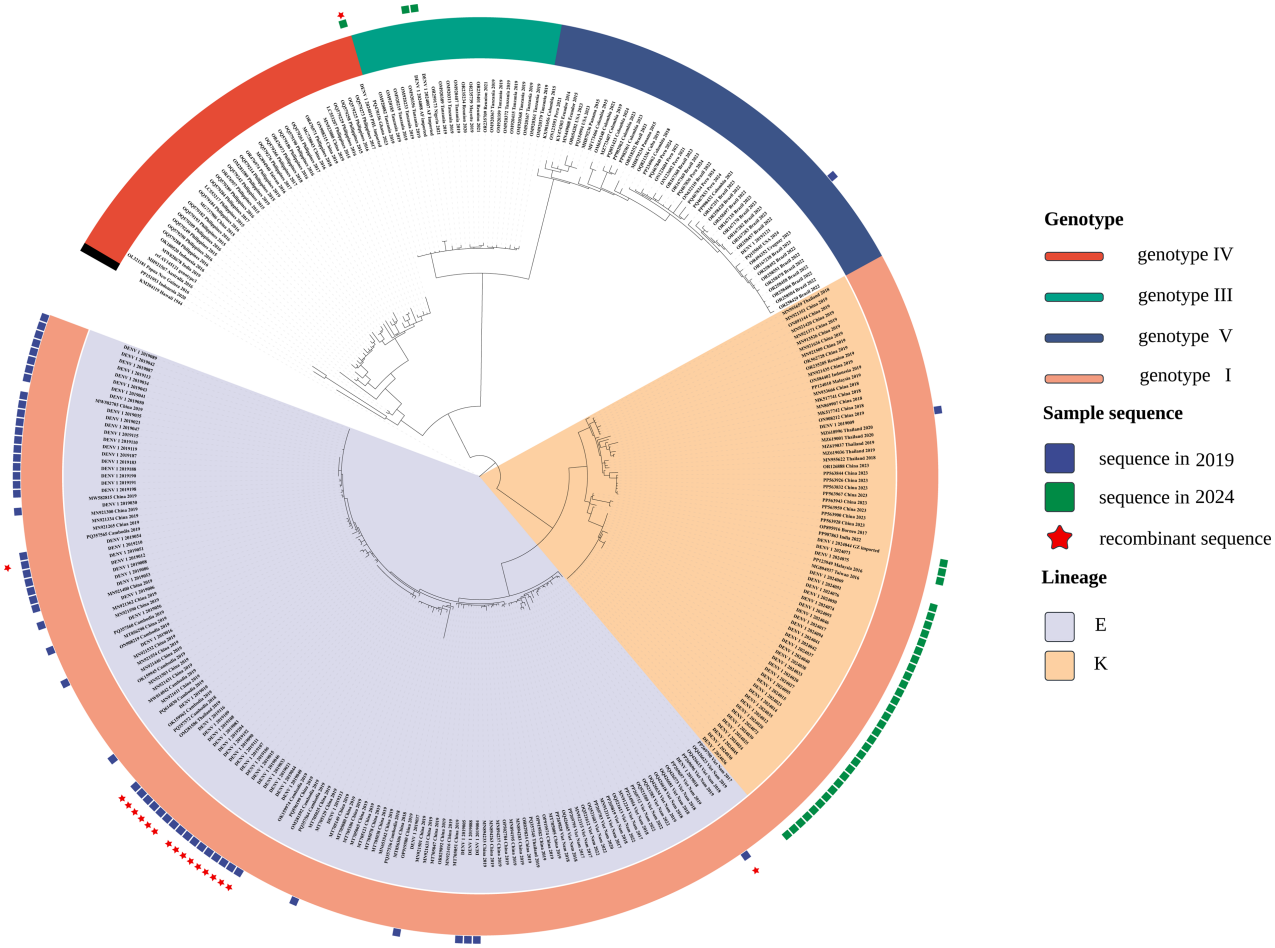
Focused phylogenetic tree depicting the evolutionary relationships among DENV-1 from local samples—56 isolates from 2019 (blue squares) and 37 from 2024 (green squares)—alongside closely related reference strains.

Phylogenetic reconstruction indicated that DENV-1 isolates from 2019 and 2024 were assigned to genotype I but segregated into distinct lineages, implying divergent origins (Figure 1). To enhance interpretability and emphasize lineage-level relationships, a pruning strategy based on MRCA inference was employed. For each local isolate, only reference sequences sharing an MRCA node with fewer than 50 terminal taxa were retained, reflecting close phylogenetic relatedness. This threshold was calibrated to preserve analytical specificity while maintaining sufficient evolutionary context. The resulting focused tree (Figure 1) delineated sequences with high genetic similarity to the regional isolates.

The 2019 isolates predominantly aligned with the 1I_E.1 lineage, clustering alongside sequences from Guangzhou, Fujian, Nanchang, Shenzhen, Shanghai, and Cambodia. In contrast, 2024 samples primarily associated with the 1I_K.2 lineage, indicating a potential lineage shift over time. Temporal clustering patterns, particularly the close phylogenetic associations with Cambodian and Vietnamese strains, suggest distinct viral introduction events across outbreak periods and inform downstream recombination analysis.

### 3.2 Selection pressure and recombination analysis

Codon-level selection analysis indicated uniform purifying selection across the entire DENV-1 coding region, reflecting strong evolutionary constraints that preserve genomic stability and functional integrity of key viral elements.

Eighteen statistically supported recombination events were identified among regional sequences, revealing two distinct recombination configurations (Supplementary Table S1). The first and more prevalent pattern was detected in 17 regional isolates collected in 2019, involving recombination between HM631851 and KF955446, both Vietnamese strains from 2008, with a recombinant fragment extending from nucleotide positions 95 to 4,209. This region includes the complete set of structural proteins (C, prM, E) and the early non-structural protein NS1. The second, less frequent pattern appeared in a single imported case from the Philippines (DENV-12024019), involving ON911333 (a Cambodian strain from 2019) and an uncharacterized parental lineage, with distinct recombination breakpoints.

The recombinant segment spanning positions 95–4,209 holds particular relevance due to its inclusion of all structural protein-coding regions, capsid (C), pre-membrane (prM), and E, in addition to NS1, suggesting potential implications for antigenic variation and viral fitness.

### 3.3 Molecular evolution of DENV-1 in southeastern coastal China

**Figure 2:**
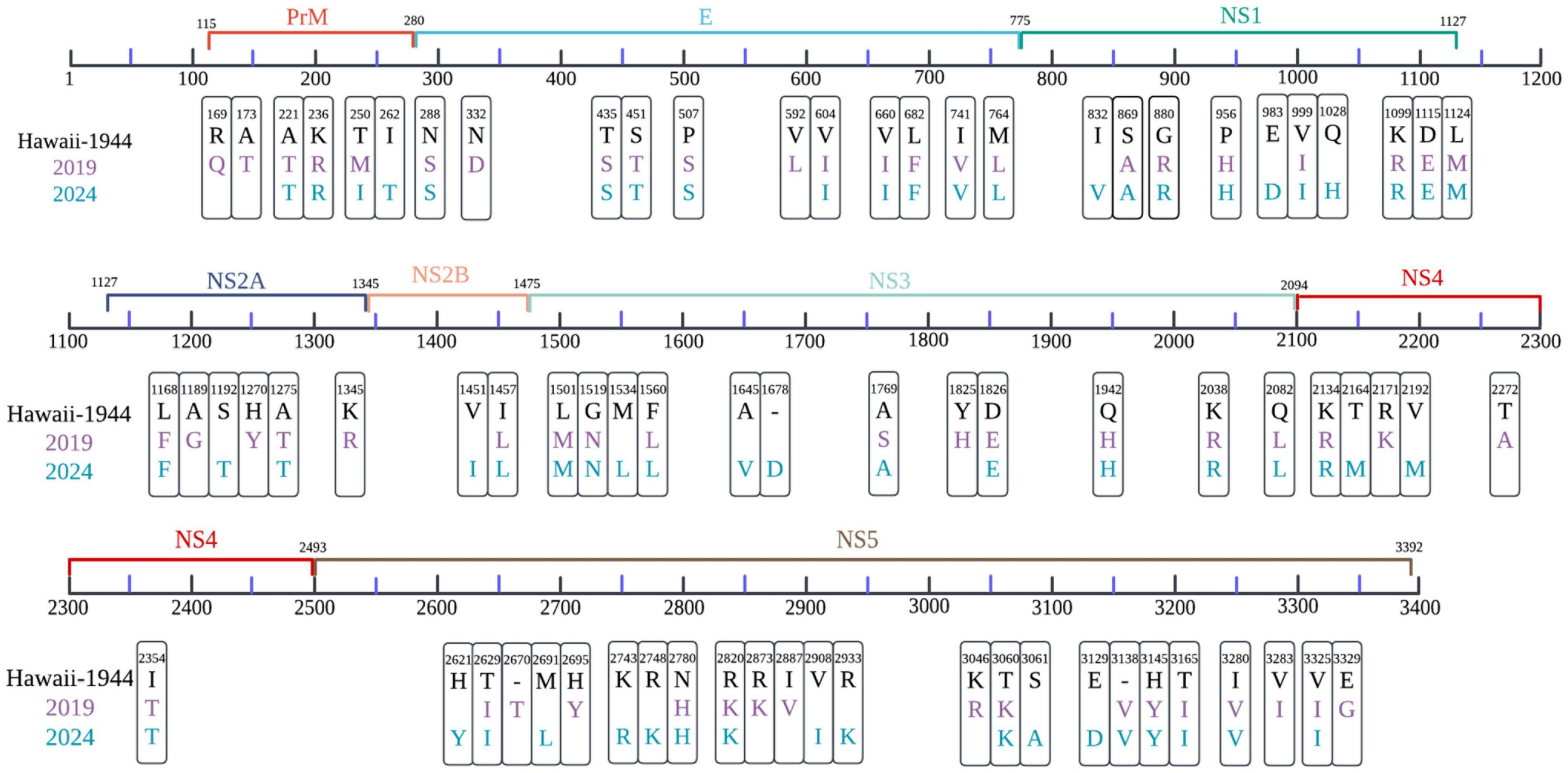
Comparative analysis of amino acid substitutions in local DENV-1 strains from 2019 (purple) and 2024 (blue), using the Hawaii strain (1944) as a reference. Non-synonymous variants across the viral polyprotein were mapped, with significant sites identified through Hamming distance calculations, applying a divergence threshold of >50% across sequences. Color coding corresponds to sampling year, and residue positions are annotated according to polyprotein coordinates.

Relative to the DENV-1 reference strain (KM204119, Hawaii-1944), samples collected in 2019 exhibited 58 non-synonymous amino acid substitutions, whereas 64 were identified in 2024 isolates, with 40 mutations conserved across both periods (Figure 2).

The prM region of 2019 samples displayed three distinct substitutions, R169Q, A173T, and T250M. In the E protein region, nine shared substitutions, including P507S and L682F.

A total of 24 substitutions were exclusive to 2024 samples and absent in 2019, primarily localized to NS3 and NS5 proteins (Figure 2), indicating continued diversification of the post-pandemic 1I_K.2 lineage.

This mutational trend parallels the observations reported by Cruz-Oliveira et al. (2024), who documented accelerated DENV-1 molecular evolution across multiple regions following the pandemic. The authors attributed the emergence of distinct mutational signatures to transmission perturbations associated with resumed human mobility, potentially creating evolutionary niches for novel variant development.

### 3.4 Comparison with vaccine strains

**Figure 3:**
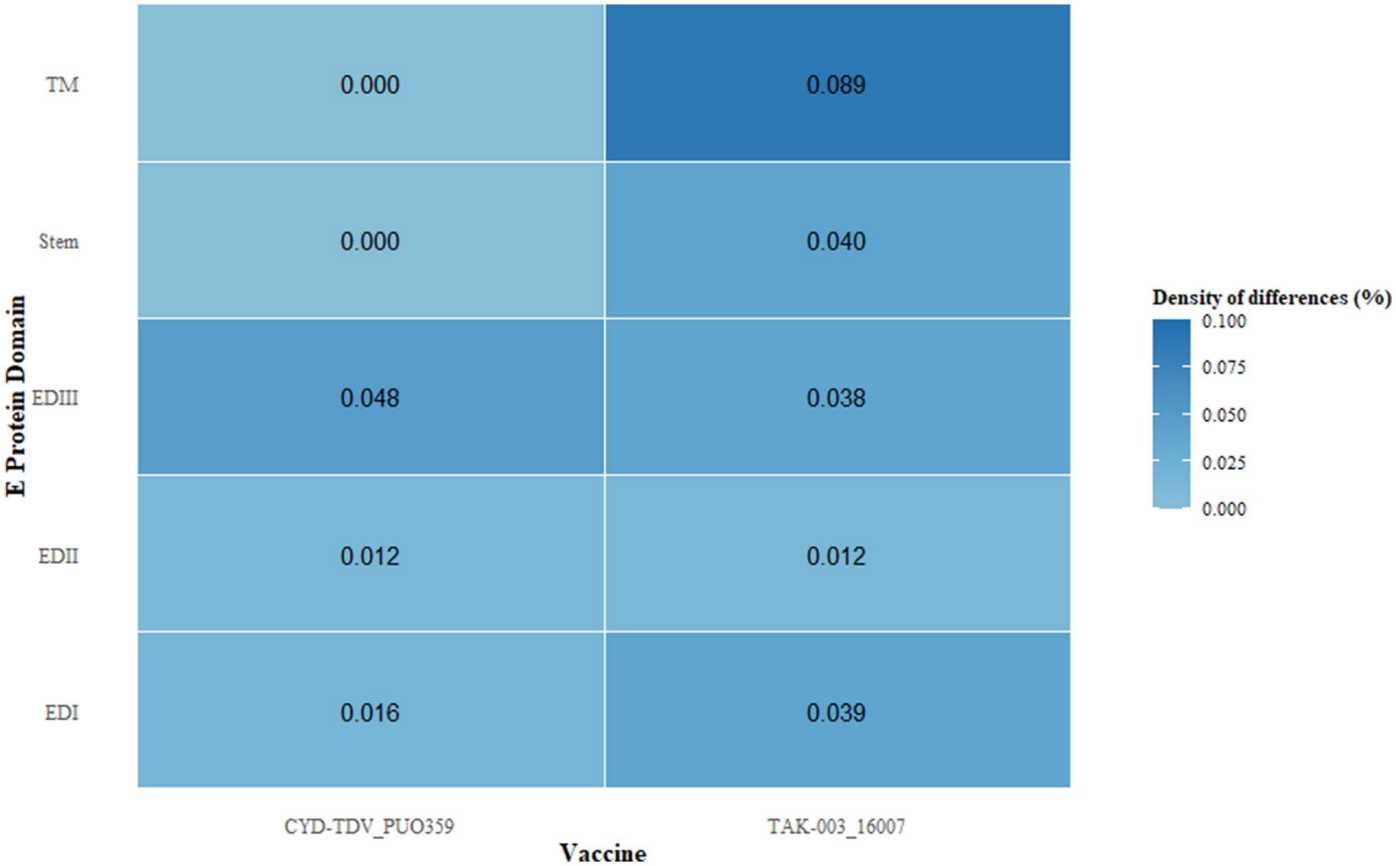
Differential density, defined as the ratio of non-synonymous substitutions to domain length, was calculated across distinct regions of the envelope protein, including domains EDI–III, the stem, and the transmembrane (TM) segments. This metric quantifies the relative accumulation of amino acid changes normalized by structural domain size.

Comparative assessment of the E protein sequences revealed antigenic divergence between regional DENV-1 strains and the vaccine strains CYD-TDV and TAK-003. In the EDIII region of CYD-TDV’s E protein, nine amino acid substitutions were detected relative to regional isolates, whereas TAK-003 exhibited 17 substitutions. Domain-specific analysis of substitution frequency across the E protein indicated a non-uniform distribution, with EDIII displaying the highest substitution density, consistent with its role in receptor engagement and its inclusion of key neutralizing epitopes (Figure 3). A substantial proportion of these substitutions were located within previously characterized epitope regions.

**Figure 4:**
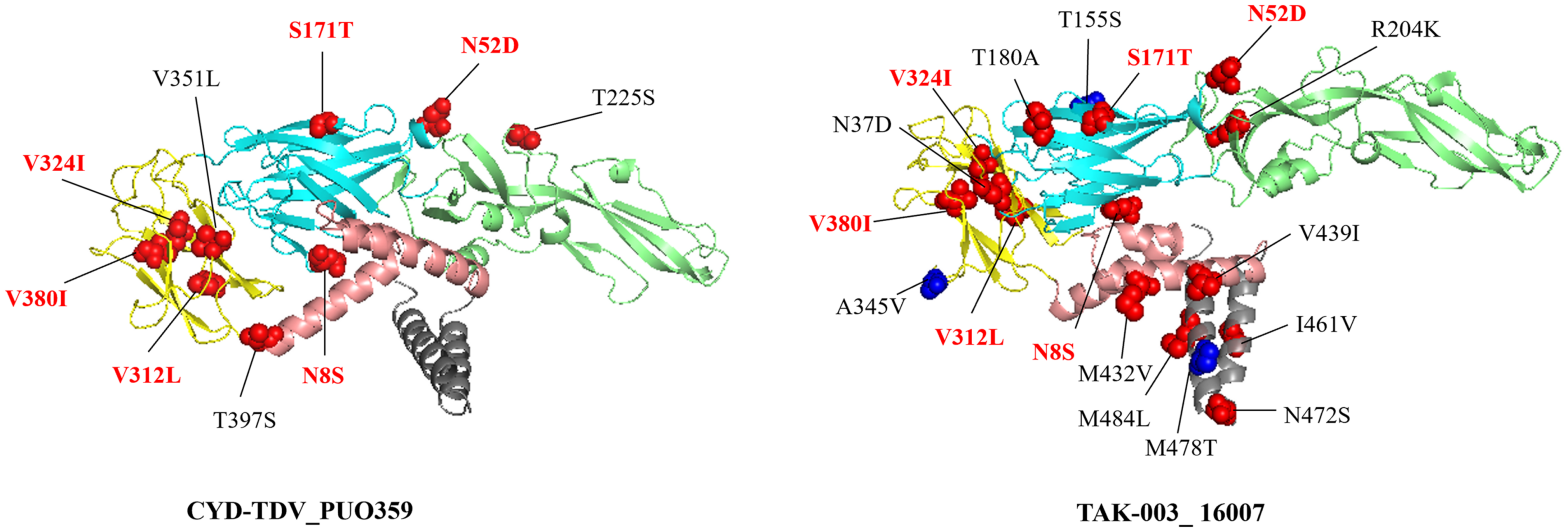
Amino acid substitutions with a frequency exceeding 50% relative to the vaccine strain are mapped onto the dimeric envelope protein structure. Mutations located within established epitope regions are highlighted in red, while those outside known epitopes are marked in blue. Residues annotated in red text (N8S, N52D, S171T, V312L, V324I, and V380I) shared mutations between the two substitutions present in both vaccine strains.

Structural projection of amino acid substitutions onto the envelope protein dimer demonstrated that multiple high-frequency variants (>50%) clustered within established epitope sites (red), while others appeared in non-epitope regions (blue) (Figure 4). Among these, six mutations (N8S, N52D, S171T, V312L, V324I, and V380I) were consistently present in both vaccine strains and regional isolates, suggesting fixation of these residues within the circulating viral population. The three-dimensional localization of these substitutions, particularly those situated in solvent-accessible areas, informs potential effects on antibody accessibility and binding dynamics.

## 4 Discussion

DENV-1 evolutionary patterns in southeastern coastal China indicate that imported cases exert substantial influence on local dengue dynamics, particularly during the COVID-19 pandemic. Interrupted time series analysis identified an upward trend in dengue incidence following the relaxation of COVID-19 restrictions in December 2022 (*p* = 0.059), while case counts remained lower from January 2020 to November 2022 relative to pre-pandemic levels (Supplementary Figure S1 and Table S2). Phylogenetic analysis revealed a shift from the predominance of the 1I_E.1 lineage in 2019 to the 1I_K.2 lineage in 2024, a transition that temporally overlapped with the pandemic, though additional determinants may underlie this shift. Surveillance data from 2023 documented a rise in imported dengue cases concurrent with the appearance of the 1I_K.2 lineage. Pandemic-related global mobility disruptions (Koutsakos et al., 2021) offered a distinctive framework for interpreting dengue transmission patterns. In the absence of continuous viral strain introductions, local transmission networks markedly diminished, indicating a dependency on intermittent importation events to sustain transmission. This transmission profile corresponded with patterns observed in other Chinese provinces, where DENV-1 outbreaks were driven by recent external introductions (Ma et al., 2021; Liu et al., 2022; Guan et al., 2021; Sheng et al., 2022). Although interventions such as mask-wearing primarily targeted respiratory pathogens (Leung et al., 2020), constraints on human movement likely played a more substantial role in limiting dengue transmission by reducing opportunities for new strain introduction. The pandemic thus functioned as a quasi-natural experiment, illustrating that sustained endemic circulation is unlikely without ongoing importation, reinforcing (Wu et al., 2010) characterization of dengue in China as an imported disease, with significant implications for the design of surveillance and intervention strategies.

At the molecular level, non-structural protein 1 (NS1) elucidates the mechanistic basis of the transmission pattern through its integral involvement in viral replication and modulation of host immune responses (Fisher et al., 2023). Functioning as a multifunctional component of the viral machinery, NS1 contributes to replication processes and serves as a diagnostic marker with clinical relevance (Muller and Young, 2013). Recombination analysis in this study revealed events localized within the NS1 region, particularly among 2019 isolates exhibiting breakpoints between nucleotide positions 95 and 4,209, spanning structural proteins and NS1. These patterns predominantly involved Vietnamese and Cambodian parental strains (HM631851 and KF955446), implicating transnational genetic exchange. Such recombination events may enhance viral adaptability and fitness, with potential implications for transmission dynamics and disease outcomes. Clarification of their epidemiological impact requires additional functional analyses. The results support the existence of DENV-1 genetic exchange networks across Southeast Asia and delineate evolutionary linkages between epidemic strains from southeastern coastal China and those circulating in proximate regions such as Vietnam and Cambodia, thereby furnishing molecular evidence consistent with an importation-driven transmission model.

Beyond recombination dynamics, the antigenic divergence between regional strains and commercial vaccines highlights intricate evolutionary interactions, particularly within the EDIII domain. Comparative analysis showed that 2019 and 2024 isolates carried 58 and 64 non-synonymous amino acid substitutions, respectively, relative to the DENV-1 reference, with 40 mutations conserved across both timepoints. The 24 substitutions exclusive to 2024 were primarily localized to NS3 and NS5 proteins, suggesting continued molecular adaptation within the post-pandemic 1I_K.2 lineage. CYD-TDV exhibited closer antigenic alignment with regional strains than TAK-003; however, prior studies have demonstrated that even limited antigenic divergence can reduce vaccine-induced protection (Katzelnick et al., 2015; Gallichotte et al., 2015). This mutational trajectory mirrors reports of intensified evolutionary rates following pandemic-associated perturbations (Cruz-Oliveira et al., 2024). The functional implications of NS3 and NS5 substitutions remain to be elucidated through targeted experimentation, particularly in relation to viral replication dynamics, fitness, and virulence. In light of this antigenic variability and its prospective consequences for vaccine performance, sustained region-specific genomic surveillance and incorporation of prior immunity profiles are critical considerations for optimizing immunization strategies.

By synthesizing epidemiological trends, molecular signatures, and antigenic profiles, this study offers a structured framework for dissecting, monitoring, and managing DENV-1 transmission within Southeast coastal regions. Comparative analyses of regional DENV-1 strains revealed distinct molecular replacement patterns, while key amino acid substitutions were evaluated to support lineage tracking. Compatibility with existing vaccine strains was examined to inform localized intervention strategies. Molecular data were aligned with epidemiological metrics to enable an integrated surveillance approach. Collectively, the findings provide a foundational basis for refining DENV-1 control efforts in coastal settings. Limitations include a relatively limited sample size, absence of associated clinical metadata, reliance in silico methodologies, and potential bias introduced by temporal confounders in longitudinal analyses. Further investigations should broaden sample representation, incorporate clinical datasets, and experimentally verify high-impact substitutions, particularly within NS3 and NS5 regions, to elucidate their functional roles and biological consequences. Such efforts would enhance understanding of how imported infections influence transmission dynamics and inform targeted prevention strategies.

Computational analysis revealed distinct amino acid variations in the E protein, particularly within the EDIII domain, when comparing regional DENV strains to vaccine strains. Although such differences may imply antigenic divergence, predictions derived from in silico methods are inherently constrained and necessitate validation through neutralization assays. Notably, clinical efficacy of dengue vaccines is governed by a complex interplay of factors beyond antigenic similarity. For instance, Sridhar et al. (2018) reported that serostatus prior to vaccination markedly influenced protection levels, with 74% efficacy observed in seropositive individuals versus 38% in seronegative counterparts (*p <* 0.001). In light of the observed sequence divergence, additional studies incorporating neutralization assays using sera from vaccinated individuals and locally circulating strains are warranted to clarify potential implications for vaccine effectiveness in this setting. Concurrently, dengue mitigation strategies should maintain focus on established public health measures, including vector control and rigorous surveillance of imported infections, in alignment with WHO recommendations (World Health Organization, 2018).

In conclusion, whole-genome sequencing revealed a complete shift in circulating DENV-1 strains in southeastern coastal China, transitioning from the 1I_E.1 lineage in 2019 to the 1I_K.2 lineage in 2024. This turnover involved 40 shared non-synonymous substitutions across the two periods and 24 substitutions unique to the 2024 isolates, predominantly located within NS3 and NS5 coding regions. Comparative analysis of regional strains and commercial vaccine strains identified amino acid mismatches in the E protein, including 9 in CYD-TDV and 17 in TAK-003, with all variants situated within previously characterized epitope regions. These observations warrant further experimental validation to assess potential impacts on vaccine-induced protection in this setting. The timing of strain replacement overlapped with COVID-19-related disruptions; however, additional contributing factors likely include intrinsic viral evolutionary dynamics and altered patterns of human mobility. Phylogenetic clustering of 2019 and 2024 isolates with strains from adjacent countries, along with recombination signals involving Cambodian and Vietnamese parental lineages, supports the hypothesis that regional outbreaks are primarily initiated by viral introductions followed by localized transmission. This updated characterization of DENV-1 dynamics in a non-endemic context refines current understanding of regional viral evolution, outlines challenges for vaccine performance, and reinforces the necessity for continuous molecular surveillance and geographically tailored control strategies in southeastern coastal China.

## Data Availability

The datasets presented in this study can be found in online repositories. The names of the repository/repositories and accession number(s) can be found at: https://www.ebi.ac.uk/ena, PRJEB88166.
