# Supplementary material for: The pre- and post-COVID-19 pandemic dengue fever patterns in southeastern coastal China in 2019 and 2024: molecular evolution and strain replacement
Source: Front Microbiol. 2025 Aug 8;16:1607085. doi: 10.3389/fmicb.2025.1607085 (PMC12371238; doi:10.3389/fmicb.2025.1607085)
Supplement: Supplementary file 1 [file Table_1.docx]

**Table S1: Recombination analysis of DENV-1 by RDP**"+" indicates statistically significant detection of a recombination event (p<0.05) by the corresponding algorithm; "–" denotes the absence of detectable recombination events by that specific method.

| Recombinant sequence | Break point position | Parental sequence | Detectionmethods | | | | | | |
| --- | --- | --- | --- | --- | --- | --- | --- | --- | --- |
|  | Begin/End | Major/Minor | RDP | GENECONV | BootScan | Maxchi | Chimaera | SiScan | 3Seq |
| DENV_1_2019015 | 95/4209 | HM631851/KF955446 | + | + | - | + | + | + | + |
| DENV_1_2019021 | 95/4209 | HM631851/KF955446 | + | + | - | + | + | + | + |
| DENV_1_2019040 | 95/4209 | HM631851/KF955446 | + | + | - | + | + | + | + |
| DENV_1_2019046 | 95/4209 | HM631851/KF955446 | + | + | - | + | + | + | + |
| DENV_1_2019106 | 95/4209 | HM631851/KF955446 | + | + | - | + | + | + | + |
| DENV_1_2019108 | 95/4209 | HM631851/KF955446 | + | + | - | + | + | + | + |
| DENV_1_2019111 | 95/4209 | HM631851/KF955446 | + | + | - | + | + | + | + |
| DENV_1_2019192 | 95/4209 | HM631851/KF955446 | + | + | - | + | + | + | + |
| DENV_1_2019210 | 95/4209 | HM631851/KF955446 | + | + | - | + | + | + | + |
| DENV_1_2019018 | 53/4209 | HM631851/KF955446 | + | + | - | + | + | + | + |
| DENV_1_2019033 | 95/4209 | HM631851/KF955446 | + | + | - | + | + | + | + |
| DENV_1_2019044 | 95/4209 | HM631851/KF955446 | + | + | - | + | + | + | + |
| DENV_1_2019083 | 95/4209 | HM631851/KF955446 | + | + | - | + | + | + | + |
| DENV_1_2019107 | 95/4209 | HM631851/KF955446 | + | + | - | + | + | + | + |
| DENV_1_2019109 | 95/4209 | HM631851/KF955446 | + | + | - | + | + | + | + |
| DENV_1_2019116 | 95/4209 | HM631851/KF955446 | + | + | - | + | + | + | + |
| DENV_1_2019204 | 95/4209 | HM631851/KF955446 | + | + | - | + | + | + | + |
| DENV_1_2024019 | 464/1136 | unknown/ON911333 | + | + | + | + | + | + | + |
