# Supplementary material for: The pre- and post-COVID-19 pandemic dengue fever patterns in southeastern coastal China in 2019 and 2024: molecular evolution and strain replacement
Source: Front Microbiol. 2025 Aug 8;16:1607085. doi: 10.3389/fmicb.2025.1607085 (PMC12371238; doi:10.3389/fmicb.2025.1607085)
Supplement: Supplementary file 2 [file Table_2.docx]

**Table S2: Monthly dengue fever case counts and segmented regression analysis, 2018-2024**

number of cases：

|  | time | number of cases |
| --- | --- | --- |
| 1 | 2018-01 | 1 |
| 2 | 2018-02 | 0 |
| 3 | 2018-03 | 0 |
| 4 | 2018-04 | 1 |
| 5 | 2018-05 | 0 |
| 6 | 2018-06 | 0 |
| 7 | 2018-07 | 0 |
| 8 | 2018-08 | 1 |
| 9 | 2018-09 | 0 |
| 10 | 2018-10 | 0 |
| 11 | 2018-11 | 0 |
| 12 | 2018-12 | 1 |
| 13 | 2019-01 | 2 |
| 14 | 2019-02 | 0 |
| 15 | 2019-03 | 0 |
| 16 | 2019-04 | 0 |
| 17 | 2019-05 | 3 |
| 18 | 2019-06 | 4 |
| 19 | 2019-07 | 4 |
| 20 | 2019-08 | 24 |
| 21 | 2019-09 | 102 |
| 22 | 2019-10 | 3 |
| 23 | 2019-11 | 3 |
| 24 | 2019-12 | 2 |
| 25 | 2020-01 | 0 |
| 26 | 2020-02 | 0 |
| 27 | 2020-03 | 0 |
| 28 | 2020-04 | 0 |
| 29 | 2020-05 | 0 |
| 30 | 2020-06 | 0 |
| 31 | 2020-07 | 0 |
| 32 | 2020-08 | 0 |
| 33 | 2020-09 | 0 |
| 34 | 2020-10 | 0 |
| 35 | 2020-11 | 0 |
| 36 | 2020-12 | 0 |
| 37 | 2021-01 | 0 |
| 38 | 2021-02 | 0 |
| 39 | 2021-03 | 0 |
| 40 | 2021-04 | 0 |
| 41 | 2021-05 | 0 |
| 42 | 2021-06 | 0 |
| 43 | 2021-07 | 0 |
| 44 | 2021-08 | 0 |
| 45 | 2021-09 | 0 |
| 46 | 2021-10 | 0 |
| 47 | 2021-11 | 0 |
| 48 | 2021-12 | 0 |
| 49 | 2022-01 | 0 |
| 50 | 2022-02 | 0 |
| 51 | 2022-03 | 0 |
| 52 | 2022-04 | 0 |
| 53 | 2022-05 | 0 |
| 54 | 2022-06 | 0 |
| 55 | 2022-07 | 0 |
| 56 | 2022-08 | 0 |
| 57 | 2022-09 | 0 |
| 58 | 2022-10 | 0 |
| 59 | 2022-11 | 0 |
| 60 | 2022-12 | 0 |
| 61 | 2023-01 | 0 |
| 62 | 2023-02 | 2 |
| 63 | 2023-03 | 1 |
| 64 | 2023-04 | 1 |
| 65 | 2023-05 | 0 |
| 66 | 2023-06 | 0 |
| 67 | 2023-07 | 4 |
| 68 | 2023-08 | 1 |
| 69 | 2023-09 | 0 |
| 70 | 2023-10 | 0 |
| 71 | 2023-11 | 0 |
| 72 | 2023-12 | 0 |
| 73 | 2024-01 | 0 |
| 74 | 2024-02 | 1 |
| 75 | 2024-03 | 1 |
| 76 | 2024-04 | 0 |
| 77 | 2024-05 | 3 |
| 78 | 2024-06 | 3 |
| 79 | 2024-07 | 2 |
| 80 | 2024-08 | 18 |
| 81 | 2024-09 | 36 |
| 82 | 2024-10 | 15 |
| 83 | 2024-11 | 7 |
| 84 | 2024-12 | 1 |

Coefficient Estimates：

| Parameter | Coefficient | HAC Std. Error | z-value | p-value | 95% CI |
| --- | --- | --- | --- | --- | --- |
| Time trend (_t) | 1.015 | 0.733 | 1.38 | 0.166 | [-0.422, 2.452] |
| Level change at Jan 2020 (_x2020m1) | -18.982 | 13.184 | -1.44 | 0.15 | [-0.422, 2.452] |
| Trend change at Jan 2020 (xt2020m1) | -1.015 | 0.733 | -1.38 | 0.166 | [-2.452, 0.422] |
| Level change at Dec 2022 (_x2022m12) | -2.603 | 2.097 | -1.24 | 0.215 | [-6.714, 1.507] |
| Trend change at Dec 2022 (xt2022m12) | 0.537 | 0.284 | 1.89 | 0.059 | [-0.021, 1.094] |
| Intercept (_cons) | -5.383 | 4.752 | -1.13 | 0.257 | [-14.698, 3.931] |
