# Supplementary figures and images for: The pre- and post-COVID-19 pandemic dengue fever patterns in southeastern coastal China in 2019 and 2024: molecular evolution and strain replacement
Source: Front Microbiol. 2025 Aug 8;16:1607085. doi: 10.3389/fmicb.2025.1607085 (PMC12371238; doi:10.3389/fmicb.2025.1607085)

Figure S1: Interruption Time Series Analysis (2018-2024)

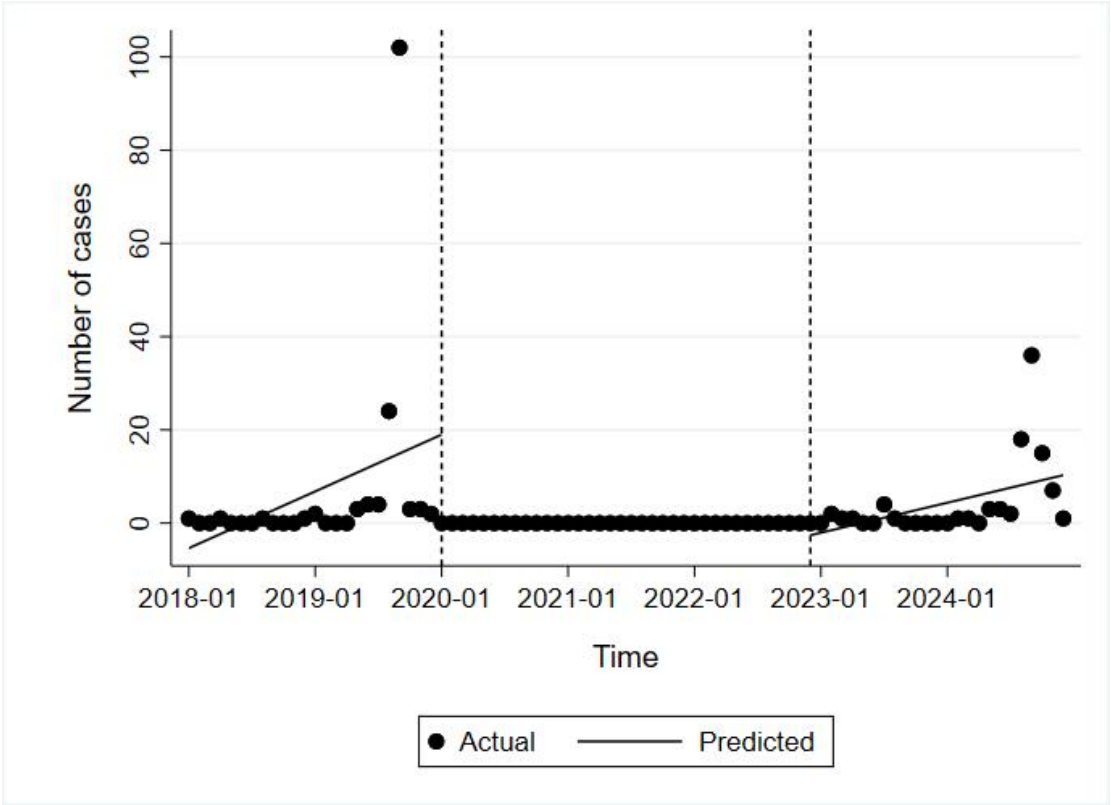

Supplement: Supplementary file 3 [file Image_1.pdf]
